# Hours of Employment: Council's Answer to Labour Minister

**Published:** 1920-12-18

**Authors:** 


### GENERAL NURSING COUNCIL FOR ENGLAND AND WALES.

### Hours of Employment: Council's Answer to Labour Minister.

The eleventh meeting of the General Nursing Council was held at the Ministry of Health on Friday, December 10. The Chairman of the Council, Mr. J. C. Priestley, K.C., was in the chair, and the following members were present: The Hon. Mrs. Eustace Hills, Rev. G. B. Cronshaw, Dr. E. W. Goodall, Sir T. Jenner Verrall, Miss A. Cattell, Mr. T. Christian, Miss A. Coulton, Miss R. Cox Davies, Miss A. Dowbiggin, Mrs. E. G. B. Fenwick, Miss A. Lloyd Still, Miss M. MacCallum, Miss I. Macdonald, Miss A. M. Peterkin, Miss E. Smith, Miss E. C. Suiss, Miss S. E. Villiers, Miss C. Worsley, and Miss Seymour Yapp. Letters of regret were notified from Lady Hobhouse, Miss Sparshott, and Dr. Bedford Pierce.

### Cottage Nurses.

A letter from the College of Nursing, dated December 2, was reported. It conveyed a resolution of a meeting of the members of the London Centre stating that in its opinion a Supplementary Register for Cottage Nurses is unnecessary, and would be a danger to the public. The Royal British Nurses' Association had sent a letter to the Council, dated December 6, containing a resolution on the same subject. It supported the General Nursing Council in its decision not to establish such a Register, and stated its opinion that "if the title 'registered' is bestowed upon cottage nurses, as such, both trained nurses and the public will be deprived of the privileges and protection to be effected through the Nurses' Registration Acts."

### Rules for Existing and Future Nurses.

The Council considered *in camera* the notes of the Minister of Health on the rules of the Council for existing nurses. When the Press returned the report of the Registration Committee as to a new rule for future nurses—*i.e.*, nurses whose training expires before July 1, 1924—was under consideration. Provision is required for such nurses pending the coming into operation of the Council's rules as to training, curriculum, etc.

Mrs. Bedford Fenwick presented the further report of the Registration Committee on the form of certificate and seal. It was decided to accept the estimate before the Council for the seal, and to await further specimens and particulars of the certificates.

### Nurses and the Hours of Employment Bill.

The Council then discussed at considerable length the reply to be sent to the letter of the Minister of Labour dated July 7. This matter has been before the October and November meetings of the Council, and at the October meeting the Council voted that nurses should be taken out of the Minister of Labour's Bill. At the November meeting of the Council a resolution proposed by Dr. Bedford Pierce was carried by 14 votes to 6 asking the Minister of Health to introduce a Bill to regulate nurses' hours, and a further resolution proposed by Miss Cox Davies was carried that in such a Bill a nurse's hours should be limited to forty-eight per week.

When the question of the reply to be sent was brought up by the Chairman at the meeting of December 10, Mrs. Bedford Fenwick remarked that these resolutions could not both stand—*i.e.*, the resolutions that nurses be taken out of the Hours of Employment Bill and that the Minister of Health be asked to bring in a Bill. She desired that nurses shall be included in the Hours of Employment Bill except private nurses, who, she said, wish to come out of the Bill. In support of this she instanced a resolution to this effect which had been passed at a meeting of nurses at the Medical Society's Room on December 6, and she repeated a medical opinion that any limitation of private nurses' hours would be unworkable.

Miss MacCallum, on the other hand, expressed her opinion that the private nurse's life is harder than that of any other, that she has no one to defend her, that the larger number of nurses in the United Kingdom are private nurses, and that it would be a very serious thing to remove them from the protection of any Employment Bill.

### Mental Nurses and the Bill.

Mr. T. Christian stated that the Association which he represented comprises seven-eighths of the mental nurses; they strongly objected to being brought into any Bill under the Minister of Health, and had urged that they be included in the Ministry of Labour's forty-eight-hour Bill. They would not be satisfied to be in a Bill of the Minister of Health, and he further took objection to what he referred to as a reservation in the Council's resolution limiting the number of hours to forty-eight—viz., the words "in attendance upon the sick." These words, he said, were a qualification which would make the limitation of hours no good to the nurses. So far as mental nurses were concerned, their work included other duties than actual nursing; mental patients were either abnormal or sub-normal, and the nurse had to live up to or down to their standard. No stretch of the imagination could consider that as anything but duty, no matter whether the duty was playing billiards or games or taking part in any other form of amusement.

Miss Cox Davies explained that it was for that very reason that the words "in attendance upon the sick" were used. She very strongly held that nurses were in attendance on the sick whatever they might be doing during such attendance.

Mr. Christian said the intention may have been good, but there were many people not actually in attendance on the sick who would not be included in a forty-eight-hour week.

### The Position to be Dealt With.

The Chairman then returned to the question of the reply to be sent to the Minister of Labour's letter, Miss Seymour Yapp stating that she was quite willing for private nurses working on their own account to be excluded from the Bill. Mrs. Bedford Fenwick submitted that the resolution to take nurses out of the Labour Bill should be rescinded. Miss Cattell desired that nurses should be left in that Bill, but the Chairman pointed to the Council's resolution. Sir T. Jenner Verrall, in a short but forceful statement, then summarised the position. The Council had taken two steps: First, by a small majority it had asked that nurses should be taken out of the Labour Bill, and, second, it had resolved to ask that nurses should be included in a Ministry of Health Bill. Some compromise must be found, and possibly would be accepted, as the majorities in both resolutions were small, but in any case an answer to the letter of the Minister of Labour was due, and he considered it important to give an answer.

Dr. Goodall pointed out that the Minister of Labour's letter of July 7 asked for a reply on two specific points: one was on the scheme put forward by the College of Nursing, though not limiting consideration to that scheme, and the second on the class of nurses to be included. What the Council had to do that day was to inform the Minister of Labour that nurses did not wish to be under his Bill, but to be in a Bill to be brought in by the Minister of Health, and it had also to state what classes of nurses it wished to be brought under that Bill.

### The Point of the College Scheme.

Mrs. Bedford Fenwick proposed that the Council should inform the Minister of Labour that it did not approve of the schedule as drafted by the College of Nursing and sent to the Minister of Labour because it asks for an additional day's labour from the nurses. This was seconded by Miss Macdonald, who remarked that in her view nurses should have a shorter working week rather than a longer. Miss Cox Davies pointed out that this was only one point in the College scheme, which contained two others. On the resolution being put to the meeting it was carried. Miss

**General Nursing Council**—(*continued*).

Cox Davies explained she did not vote, seeing that the resolution dealt only with one point in the College scheme. Miss Lloyd Still pointed out that the College scheme asked for the inclusion of nurses in a Special Order in the Hours of Employment Bill in the same way that agricultural workers were included in a Special Order under that Bill, and she suggested an analogy between the two classes of workers.

Further discussion took place, in which Dr. Goodall, the Chairman, Mrs. Bedford Fenwick, Miss Cox Davies, Miss Villiers, and Miss Seymour Yapp took part. Mrs. Fenwick asked again if anyone desired to rescind the resolution passed at the last meeting, and a question was put to the meeting by the Chairman asking if it was the Council's desire that the discussion should be postponed in order that some member might put a resolution, if desired, to rescind any resolution previously passed. Eight voted in favour and nine against any postponement.

Sir T. Jenner Verrall then proposed that an answer be sent to the letter from the Minister of Labour conveying to him the resolutions passed (1) that nurses should be excluded from the Bill now before the House connected with the Ministry of Labour; (2) that the Minister of Health should be asked to bring in a Bill as covered by the following resolutions: (*a*) that he be informed that the Council desires to reply that it does not approve of the scheme set forth by the College of Nursing; (*b*) that until the Minister of Health replies on the point by introducing a separate Bill the Council would rather not deal separately with the questions of institutional nursing, district nursing, and private work. This was seconded by Dr. Goodall. Miss Yapp said she desired to see the forty-eight-hour week resolution included. Mr. Christian, on behalf of mental nurses, asked that it should be made clear to the Minister that seven-eighths of the mental nurses do not wish to come out of the Minister of Labour's Bill.

The resolution was put to the meeting and carried, ten voting for it and five against.

The New Premises.

Sir T. Jenner Verrall then presented the report of the Finance Committee and accounts for settlement. He stated that *The Nursing Mirror* would not reduce its scale rate for advertising in favour of the General Nursing Council, and that the Finance Commitee had refused to pay this rate. His Committee had gone further into the question of the proposed new premises for the Council at York Gate, which were the property of Bedford College and could be sub-let to the Council at a rental of £325 per annum on a three, five, or seven years' lease. A sub-committee has been appointed to deal with the questions of decoration and furnishing.

The bills and claims were passed and the proposals concerning York Gate approved. The appointment of Miss G. E. Davies as registration clerk at a salary of £4 a week was reported.

